# Oesophageal Physiology Clinical Practice: A Case Study and Literature Review

**DOI:** 10.7759/cureus.95840

**Published:** 2025-10-31

**Authors:** Ismail Miah, Terry Wong, Sebastian Zeki, Jafar Jafari

**Affiliations:** 1 Gastroenterology, Guy's and St Thomas' NHS Foundation Trust, London, GBR; 2 Faculty of Life Sciences and Medicine, King's College London, London, GBR

**Keywords:** achalasia, clinical physiology, dysphagia, esophageal motility, high-resolution manometry

## Abstract

Clinical oesophageal physiology is continually evolving through technological advancements, developing hybrid metrics for advanced clinical measurements, and continuously refining the diagnostic guidelines. This has currently placed oesophageal physiology investigation in specialist clinical centres and is excluded from the standard upper gastrointestinal diagnostic testing conducted in general hospitals. Therefore, patients and clinicians in a general hospital have reduced accessibility to oesophageal physiology tests, and patient referrals to specialist centres are only made when all standard oesophageal diagnostic tests are normal or cannot explain the patient's symptoms. As oesophageal physiology is not widely performed in healthcare centres, practitioners in general hospitals may not always think of the oesophageal physiology diagnostic test in their line of investigations. This clinical case study presents a patient under the care of a general hospital who required oesophageal physiology surveillance to see the development of achalasia. The oesophageal physiology primary study diagnosed ineffective oesophageal motility in the absence of reflux disease. The study did, however, capture features that raised suspicion of achalasia developing on high-resolution manometry. The current clinical guidelines do not identify the pre-achalasia state and make no recommendation or set the clinical pathway for repeating the oesophageal physiology or considering oesophageal physiology surveillance. In unwrapping the clinical features for the achalasia development, this case study not only justifies the referral for oesophageal physiology surveillance, but it also offers a learning platform to interpret results beyond the technical finding, addresses pitfalls in the diagnostic guidelines, and introduces useful supplementary tests that can be implemented into routine practice to uncover the correct diagnosis and exclude achalasia mimicking conditions.

## Introduction

Progression in oesophageal clinical physiology practice has continually been pivoted through technological advancements [1-15] and achieving practitioner set goals to obtain more accurate physiological measurements [1,5-8,11-25]. The developments have been ongoing for approximately 70 years, with clinical technology reaching saturation in the last two decades. The focus has shifted to (i) pioneering metrics that are more accurately capturing the oesophageal physiology and (ii) continuously refining the guidelines for precision in the diagnostic outcome. Whilst the emphasis has been on the scientific development, patient-centred clinical practice can be overlooked at times, such as the need for repeating the oesophageal physiology or placing an oesophageal physiology surveillance. This report presents the clinical case study of a patient requiring multiple visits to an oesophageal laboratory to obtain the correct diagnosis. This is a potential area of clinical research that may require more attention.

Developments in oesophageal manometry

The concept of using oesophageal pressure changes (manometry) to assess oesophageal motility dates back to the 1950s [3], but the major development in the concept was first observed in the 1970s [3,11]. This was achieved by using external volume-displacement pressure transducers with a pneumo-hydraulic pump to drive degassed water through multiple capillary channels within the manometry catheter (these channels terminate at side-holes along the catheter, which act as a single-point recording sites along the oesophagus) [11,26].

There were three to eight single-point pressure recording sites found along the manometry catheter, which were positioned 3-5 cm apart and positioned along the oesophageal body [3,12,27-28], and one single-point sensor positioned at the lower oesophageal sphincter (LOS) high-pressure region [26]. This was the design of the conventional manometry (CM) catheter, which recorded polygraphs of oesophageal pressure changes. The polygraph was initially printed on sheets of recording paper until computers entered healthcare services in the 1980s [11,26].

Clinical IT facilitated the CM polygraph to convert electronic signals on PC screens that offered faster signal processing and obtained more data acquisition, which showed more rapid changes in the oesophageal pressure measurements during testing [7]. There was then the development of newer, robust, and compact sensor technologies with better IT interface (water-perfused and solid-state sensors) [2,3,7,12,27]. Both technologies were generating similar data, but operator preference was for the solid-state catheter as it was more convenient to prepare, clinically use, and maintain [2,3]. Solid-state technology also eliminated the possibility of artefacts being recorded from air bubbles moving within channels with water in the water-perfused catheters [2,7].

These newer sensors also highlighted a shortfall with using a single-point pressure sensor to measure the LOS function, which can arise from a misalignment of the sensor from the LOS region [26] during swallowing and even during deep respiration. This temporary misalignment of the sensor from the LOS high-pressure zone can be falsely perceived as LOS relaxation. This problem has been tackled by incorporating the Dentsleeve (Mui Scientific, Mississauga, Canada) technology into the CM, which was composed of closely packing five or six point-sensors within a 5 cm strip that was positioned along the LOS muscle length [12]. The Dentsleeve was able to capture the full length of the LOS and the high-pressure zone during head movements and swallowing manoeuvres [12,23]. Practitioners were able to then accurately obtain measurements of a weak anti-reflux barrier to explain pathological reflux or impaired LOS relaxation to diagnose achalasia.

In the early 1990s, Clouse and Staiano investigated the oesophageal body motility from swallows whilst conducting a 1 cm interval pull-through, and they identified a separation in the continuity of peristalsis in the proximal oesophagus from the mid/distal oesophagus [10]. Nowadays, this is known as the transition zone (TZ), which separates the oesophageal motility of the striated muscle and smooth muscle [17,21,23,24]. The second significant finding was the oesophageal contractility separation in the distal oesophagus [10], which is now referred to as the contractile deceleration point (CDP) [1,17,21,24]. This work in the early 1990s by Clouse and Staiano indicated the need to increase the number of sensors along the CM catheter, which would uncover hidden abnormalities that may exist between the spaced sensors (i.e., large TZ or peristaltic defects, peristatic break from diverticulum, areas of simultaneous contractions, localised hypercontractility or stricture) [8,10,24].

By the late 1990s, manometry was being performed using catheters with 21 pressure sensors, and findings were promising [2,8] that also explained the early work by Clouse and Staiano [10]. Dr Clouse found a key collaborator who formed Sierra Scientific Instruments company, and they heralded the development of the 36-sensor flexible solid-state catheter (4.2 mm diameter) in the new millennium (the product was based on a patent) [1,2,7,23,29]. The spacing between sensors in the new catheter was 1 cm, which is a small gap between sensors that could be easily interpolated without significant loss of the contractile information, and the data could be captured and displayed in real-time [7,8,10].

The oesophageal contractions detected by the sensors were transformed into spectral colours that meshed into islands of pressure from the oesophageal motor function. This became the new format for measuring the oesophageal body motility and was referred to as Clouse pressure topography. The Clouse pressure topography laid the foundation for high-resolution manometry (HRM), which was found to increase the sensitivity for detecting achalasia [3,26]. This can be seen by comparing the accuracy of the LOS relaxation recording on CM (with and without sleeve along the LOS muscle) and HRM. Pandolfino et al. reported the sensitivity of detecting achalasia on CM (with point sensor technology) and CM (with sleeve sensor technology) to be respectively 52% and 69%, and the HRM significantly increased the sensitivity detection of achalasia to 97% [24].

HRM also permits subtyping the achalasia disease stage [17,21, 24, 30] and enables distinguishing achalasia from rumination syndrome [19]. Unlike the CM screening, HRM provides simultaneous visualisation of the entire oesophageal body contractility (proximal, mid and distal segments) [7,23,24], permits mapping the functional anatomy of the striated and smooth muscle regions of the oesophagus [23] and has the ability to detect higher prevalence of gastro-oesophageal junction (GOJ) obstruction, hypomotility disorders and even upper oesophageal sphincter (UOS) disorder [26]. HRM can also capture the GOJ morphology, unlike CM, and successfully distinguish the LOS tone pressure from crura diaphragmatic indentations [1,7,16,20,23,26]. The pressure separation of the two on HRM is diagnostic of manometric hiatus hernia [7,24]. Lastly, HRM can capture oesophageal shortening phenomena occurring [24,26] and the transient LOS relaxation events [20], which are falsely perceived as LOS relaxation on CM. 

In the HRM investigation, the distal contractile integral (DCI) was developed to measure the oesophageal motor function [1,17,21,23,24]. This hybrid metric is a product of oesophageal smooth muscular contractility vigour, contractility duration, and the contractility propagation length along the oesophageal body that could be identified within the 20 mmHg isobaric contour [1,17,21,24]. DCI is assessed on standard 5 ml water swallows [17,20,21,24], which generally increases on multiple water swallows (MWS) and on solid bolus swallows (SBS) [17,20,23]. Clinical guidelines have established the DCIs on the standard 5 ml water swallows and distinguish failed peristalsis (DCIs <100 mmHgscm), weak peristalsis (100 mmHgscm <DCIs <450 mmHgscm), normal peristalsis (450 mmHgscm <DCIs <8000 mmHgscm), and hypercontractility/Jackhammer oesophagus (DCIs >8000 mmHgscm) [17,21,24]. The peristaltic contractility speed and rapidness, and the premature/spastic contractions can be assessed by the oesophageal distal latency (DL) time, which is the duration between UOS relaxation and the CDP [17,21,24].

The LOS opening is a composite measurement of the relaxation residual pressure and time, and is called the integrated relaxation pressurisation (IRP). IRP is currently based on the four-second timeframe of the lowest mean axial pressures, which is computed during standard 5 ml water swallows [1,17,20,21,23,24]. IRP can also be calculated during the rapid water drinking challenge (RDC) [17,20] and SBS [17,20,23]. The threshold for normal LOS relaxation varies, and the IRP range is currently set at <12 mmHg [17] from previously <15 mmHg for non-relaxation [21,24]. These clinical metrics, alongside recognition of Clouse topographic pressure patterns, allow interpretation for diagnosing and have led to the standardisation of HRM studies (Chicago Classification (CC)) [17, 21, 24]. The CC guidelines were created based on the HRM system developed by Sierra Scientific Instruments Inc., Los Angeles, California, United States (now under Medtronic plc, Galway, Ireland) [7,29]. 

The normative data derived for the CC editions were from using the Manoscan^TM^ 36-channel circumferential solid-state catheter and analysing the study using Manoview^TM^ imaging software (Medtronic plc) [1,7,17,21,23]. The numerical cut-offs for defining normality for IRP seem to slightly differ between HRM systems (i.e., Medtronic vs. Laborie, Orangeburg, New York, United States/Diversatek Healthcare, Milwaukee, Wisconsin, United States) [17,29]. However, principles of analysing the pressure topography plots are generalised to all HRM systems. The CC editions have been consistent in the diagnostic algorithm for a hierarchical approach in prioritising disorders of GOJ outflow obstruction and achalasia before the major disorders of peristalsis in the oesophageal body (absent contractility, distal oesophageal spasm, hypercontractile oesophagus), and lastly minor disorders of peristalsis such as ineffective oesophageal motility (IOM) [17,21,23,24].

The standard 5 ml water swallow is a small volume of liquid to initiate a swallow and evaluate the oesophageal body motility. Although this is achieved from the standard 5 ml water swallow, it does not replicate the natural physiology of people drinking water (which is by multiple swallows) and eating solid food. In addition, the standard 5 ml water swallows do not always induce or explain the patients' symptoms (dysphagia, odynophagia, non-cardiac chest pain, etc.) [23]. The latest edition of CC (version 4.0) addressed this and recommends comprehensive swallow assessments of standard 5 ml water in the upright and supine positions, and provocative testing with MWS, RDC, and SBS [17]. Bolus consistency has shown differences in the oesophageal motor function [17,23,28]. Solid bolus swallows revealed a smaller TZ, more vigorous DCI, and increased IRP compared to liquid swallows in individuals [23]. Swallows of semisolids (applesauce) also invoked higher contraction amplitudes than observed on standard 5 ml water swallows during CM, and the oesophageal transit was also reported to be longer [28].

Despite the contractility increasing with semi-solids and solids swallowing, the manometry sensor cannot measure the oesophageal transit or the bolus transport (the patient’s symptoms of dysphagia are caused by poor oesophageal transit and not necessarily from dysmotility). Patients would therefore require follow-up referral for a barium swallow (BS) study with X-ray imaging to visualise the oesophageal transit and the clearance. This led to combining the impedance sensors with the CM and HRM catheters, and this permitted measuring the oesophageal transit in parallel to oesophageal contractile propulsion forces for the bolus transport [1,3,28]. The combined CM with impedance was more sensitive to assess oesophageal function than CM alone [28], as was HRM with impedance, being more sensitive to capture poor bolus clearance than HRM alone [1].

The principle of using multiple impedance for measuring oesophageal transit stems from the original work by Silny [9], who placed a series of spaced impedance sensors along the oesophageal body to measure the alternating current between impedance sensors. The closed circuit between impedance electrodes occurs when substances are in contact between two neighbouring impedance electrodes. In the empty oesophagus, there are few ions flowing between sensors whilst in contact with the oesophageal mucosa. When a bolus is present between impedance sensors, the ionic load increases, allowing better electrical conductivity between impedance electrodes, which register as low impedance compared to the mucosa. Observing the stepwise changes between impedance sensors is the measurement of bolus movement along the oesophageal body (i.e., swallows of bolus could be displayed by antegrade impedance flow between sensors) [4,5,9,28,31]. This technique was adopted to measure oesophageal transit of various liquids (water, saline, Osmolite formula), semi-solid/viscous substances (applesauce, yoghurt), and solid bolus (bread, rice) [4,5,23,28].

The use of impedance to manometry technology to measure oesophageal transit has advantages over the BS test, as patients are not exposed to radiation or need to consume barium sulfate contrast. A BS study may be contraindicated in patients who are pregnant due to the risk of birth defects or are known with precancerous Barrett’s oesophagus, presence of perforation and fistula within the gastrointestinal tract (which would risk organ leakage of the contrast). The oesophageal physiology tests are not contraindicated by these conditions. The use of impedance with HRM is, however, relatively new to clinical practice and requires standardising the technique protocol. This includes the interpretation of impedance relationship with manometry during swallows, new classifications for diagnosis, and possibly an algorithm in the hierarchy of diagnosis. To date, the gold-standard test for diagnosing achalasia is currently with manometry and not based on the oesophageal transit with impedance transit or BS transit study.

The oesophageal contractility measuring less than 30 mmHg was originally considered hypotensive, and naturally, an isobaric contour at this magnitude was adopted for diagnostic purposes [1,24]. The significance of this is mostly to assess the TZ size and peristaltic breaks. In the first edition of CC and early years of assessing HRM topography, the peristaltic breaks were assessed at the 30 mmHg magnitude isobaric contouring [1,24]. In later editions, the peristaltic breaks were assessed at the 20 mmHg magnitude [17,21] and considered normal or small for TZ less than 3 cm in size [17,21]. The intrabolus pressurisation and pan-oesophageal pressurisation were still being measured at the 30 mmHg magnitude [17,21]. Nonetheless, the use of the isobaric contour technique to assess contractility opens a new modality of oesophageal assessment in the functional anatomy and ability to measure the functional lengths of the oesophageal muscles. Another use of the isobaric contour permits assessing the functional anatomy of the oesophagus at multiple contours (i.e., at incremental of 15 mmHg) to assess the degree of IOM on liquid and solid swallows, as well as the GOJ tone and relaxations during the swallows. This concept requires further research to evaluate its clinical significance.

Developments in gastroesophageal reflux monitoring

The discovery of oesophagitis in 1948 [32] initiated the need to measure gastric reflux and the oesophageal acid exposure time (AET) to diagnose gastrooesophageal reflux disease (GORD). The earliest studies exploring this were in 1960 by Tuttle and colleagues [33], who discovered a gastroesophageal pH gradient between the oesophagus and the stomach. Within four years, continuous recording of the oesophageal pH was possible for 12 hours, but this method required patients to immerse their hands in saline for pH reference [25]. By the 1970s, Johnson and DeMeester developed an external reference electrode that permitted the continuous measurements of oesophageal pH of the distal oesophagus for 24 hours [13]. This catheter-based pH (C-pH) probe quickly became the gold-standard diagnostic test for measuring pathological reflux and diagnosing GORD in patients [13,18,31,34].

Oesophageal pH monitoring research took another 10 years to transform into clinical practice [3], and further developments of the C-pH probe were made by having dual pH sensors to simultaneously measure pH of the distal and proximal oesophagus and even pharyngeal pH [22]. Manufacturers of the C-pH probe were flexible in catering to the end-user needs to position two pH sensors along the catheter to measure pH of the pharynx, proximal oesophagus, mid oesophagus, distal oesophagus, and stomach [5,22]. A pH sensor in the stomach permits gastric pH monitoring [5], which can be used to investigate for achlorhydria, atrophic gastritis, or therapeutic response to proton pump inhibitor (PPI) therapy, and to assess the degree of gastric acid suppression [16,22].

The pioneering of the multiple impedance sensors by Silny [9] crept into reflux monitoring studies, and by the new millennium, multiple impedance sensors were integrated into the dual C-pH probe to create the multichannel impedance-pH (MII-pH) catheter [5,28]. The impedance sensors measured changes in the alternating current between the oesophageal mucosa and the presence of substances in the oesophagus (ie, bolus, acid, etc.) [5,9,31,34]. The presence of multiple impedance sensors measured the directional flow of substance in the oesophagus. This allows distinguishing between swallows of acidic bolus from the antegrade impedance flow [4,5,9,31] and genuine gastric reflux (of both acid and non-acid) that are presented by retrograde flow on the impedance sensors [16,22,31,34]. This would eliminate capturing false-positive reflux events in the AET, which was traditionally a shortfall in the C-pH sensor recording. Liu et al. found the bolus transit on impedance sensors to be statistically longer in GORD patients compared to non-GORD patients [1]. This is interesting, with the potential for impedance sensors to screen for mechanisms of the pathological reflux (i.e., GORD from poor acid clearance or an increased number of short-lived acid reflux episodes).

The directional flow captured by impedance sensors, particularly antegrade, during MII-pH studies also opens the possibility of measuring oesophageal transit during reflux monitoring [5]. The MII-pH catheter has eight impedance sensors that are sparsely positioned along the oesophagus (17 cm, 15 cm, 9 cm, 7 cm, 5 cm, and 3 cm above the GOJ). So, currently, the impedance sensors are arbitrarily measuring impedance at locations in the oesophagus, which is similar to a CM catheter measuring mean wave amplitude (MWA) of pressures along the oesophageal body. Following the principles of creating HRM from CM by increasing the pressure sensors (from 8 to 36), the increase of impedance sensors from the current eight impedance sensors may also permit better and accurate measurements of the oesophageal impedance tomography when investigating oesophageal transit.

In parallel to the scientific advances of the catheter-based reflux monitoring technology, which poses discomfort and lifestyle restrictions during the investigation that in turn affects the study test results [35], researchers were also developing a catheter-free wireless pH (W-pH) monitoring technique from the 1960s, which was using radiotelemetry communication of pH data [14,15]. The W-pH monitoring technique, which involved clipping the pH capsule to the intraoesophageal mucosa to capture oesophageal pH data that is transmitted to a recorder, did not receive FDA approval until the 21st century [6]. By 2000, both MII-pH and W-pH reflux monitoring methods were in routine clinical practice. MII-pH method permitted ambulatory measurements of acid and non-acid refluxes, extent of reflux to mid and proximal oesophagus, assessment of true and false reflux events, oesophageal transit measurements, and the chemical clearance of acid [3,5,6,9,16,22,28,31,34]. The W-pH method provided prolonged measurements of oesophageal pH without the restriction imposed on patients having the catheterization, and prolonged oesophageal pH monitoring allowed practitioners to conduct on and off PPI therapy studies in a single test (48 hours, 96 hours) [3,16,18,36,37]. Interestingly, the pathological reflux recording on the C-pH study originally conducted by Johnson and DeMeester in 1974 (AET cut-off threshold >4.3% for pathological reflux) [13] was very similar to the average cut-off threshold that was recently found on the W-pH recording for four days (AET threshold >4.65% for pathological reflux on 96-hour monitoring) [18].

The prolonged reflux monitoring with W-pH improved the diagnostic yield of GORD and the reliability of reflux studies in clinical practice [36]. However, flaws in the W-pH study do exist from early detachment of the pH capsule, incorrect positioning of the pH capsule, and recorder-pH capsule signal loss [6,18,36]. Therefore, the cumulative reference ranges and diagnostic thresholds for 24 hours, 48 hours, 72 hours, and 96 hours were established after two decades of W-pH monitoring in clinical practice [18]. On W-pH investigations, 24-hour recordings were captured in 96% of cases, and 48-hour recordings could be captured in 89% of patients [6], and 57.4% completed >92 hours of W-pH recording [18]. Despite day-to-day variation in reflux captured in the four days of recording, the cut-off threshold for pathological reflux in 96 hours synergised with the original proposal by Lyon Consensus for MII-pH study, which was an expert opinion that has not been tested prospectively [20]. There are no parallel studies comparing GORD outcomes based on catheter-based pH studies versus W-pH studies with respect to treatment. Prolonged C-pH or MII-pH monitoring diagnostic outcome has not been compared with W-pH monitoring. Nonetheless, the current technologies facilitate a method for measuring oesophageal pH and objectively diagnose GORD. These reflux monitoring technologies also permit screening patients’ symptom correlation to reflux events, which are quantified by the symptom index (SI) and symptom association probability (SAP). SI is the fractional percentage of reflux-related symptom episodes, which is positive for >50%. SAP uses statistical cross-tabulation for reflux occurrence and symptoms in a two-minute timeframe of the pH recording. The four possible combinations are positive when the *p*-value of Fisher's exact test corresponds to a <5% chance. In other words, SAP is positive when satisfying the mathematical inequality (1-*p* value) >95% [16,20].

Research practitioners have also developed and used oesophageal impedance metrics to better understand and assess the pathophysiology of oesophageal acid exposure. This includes the mean nocturnal baseline impedance (MNBI) and post-reflux swallow-induced peristaltic wave (PSPW) [16,20]. The MNBI measures the oesophageal mucosal wall impedance, which is a predictive marker for acid-induced mucosal injury or endoscopic oesophagitis [16,20]. The PSPW is a reflex response to oesophageal acid reflux, which can be used to assess the oesophageal integrity for effective acid clearance prior to mucosal injury occurring. The Lyon consensus guidelines have proposed cut-off thresholds for MNBI and PSPW [16,20] and the use of SI, SAP, MNBI, and PSPW as adjunctive tests for inconclusive or borderline findings of oesophageal AET findings [16].

The intubation and catheterisation of the manometry and reflux test probes are considered as minimally invasive clinical procedures, and patients may experience similar side-effects to undertaking naso-gastric feeding tubes. Both patients and healthy volunteers were mostly able to tolerate the catheterisation and were able to drink/eat during the investigation [1,2,4,6-8,18,22,23,26-28,34]. The toleration is bearable, such that some patients had consented to repeating the catheter-based studies or having follow-up studies after successfully completing the test once [7,22,34], and the test results were reproducible [22]. There is a non-tolerance rate for the catheter-based reflux studies, which was reported for 7.70% patients (6.50% could not tolerate the intubation, 0.54% could not tolerate the catheterisation post intubation, and 0.70% could not tolerate the associated symptoms) [37]. The symptoms developed during catheterisation were cough, chest pain, and vomiting [26,37], which lasted for days in some patients [26]. There was no difference in the age and gender of patients tolerating and not tolerating the catheter-based studies, and the upper endoscopy findings were also no different between these two cohorts of patients (those tolerating and those not tolerating the catheterization) [37]. The literature documented that CM in general, including solid-state transducers and water-perfused-based catheters, was better tolerated than the HRM solid-state catheters [2,7,26].

The insertion and toleration of the endoscopic W-pH capsules were better tolerated by patients and healthy volunteers compared to the catheter-based studies for the reflux studies [6,35-37]. The non-tolerance rate for the W-pH capsule, based on patients wanting endoscopic removal of the pH capsule prior to completion of the study, was found in 4% of the cases [6]. The complications of aspiration, mucosal tears, oesophageal bleeding, and perforation are exceptionally rare with W-pH studies and have not been reported recently. In the current clinical practice, the gold-standard reflux testing methods, MII-pH and W-pH studies, have been successfully performed without complication, and patients and healthy volunteers were able to eat, drink, sleep, and attend work or educational courses during the investigations [18,22,36,37]. The literature has reported the W-pH reflux monitoring method to increase the diagnostic yield of GORD by >30% in negative MII-pH study for GORD [18,35,36]. There are no guidelines currently addressing the role of W-pH reflux monitoring as first-line investigation for suspected GORD, but there seem to be possible findings in the negative MII-pH study to justify the follow-up for W-pH study (i.e., number of acid reflux episodes exceeding 20 events in 24 hours, AET exceeding 1.70% and the MNBI less than 2300 Ω) [36] and the degree of the LOS laxity on the HRM study to be ≤8.0 mmHg [36].

Oesophageal physiology tests are specialised and are currently reserved for tertiary referral centres or specialist centres. The possible reasoning for this may include the rapid evolution in clinical technologies, frequent changes in the diagnostic guidelines, and the continual requirement to retrain staff. Such drawbacks may deter general hospitals from procuring the equipment and running the service. Furthermore, the oesophageal physiology service to be effective or successful would require offering comprehensive tests to measure the oesophageal motility and gastric reflux, as both are studied side-by-side, and the limitation of not offering one of the two can be detrimental to the service. Oesophageal physiology service can be expensive to establish, which not all hospitals are funded for or can aspire to. Lastly, oesophageal physiology training is not part of the mainstream of gastroenterology training, and to a GI fellow, oesophageal physiology may seem to be in a world of its own with colourful pressure topography images, unfamiliar wordy phrases and endless physiology metrics and initialisms (i.e. MWS, DCI, IRP, MNBI, RDC, SBS, CDP, PSPW, SI, SAP, etc.). In the current climate of clinical practice, it is unknown and unpredictable how the impact of this may have on patients who are under the care of a general hospital with limited access to oesophageal physiology service.

Oesophageal physiology is a niche field of gastroenterology with relatively fewer practitioners compared to other areas of gastroenterology. The technological advancements and developments of the diagnostic metrics to overcome the past clinical hurdles for accurately capturing the oesophageal physiology have been summarised. We describe the clinical case of a patient undergoing an oesophageal physiology study with the latest clinical technology, and highlight a new set of challenges to obtain the correct oesophageal physiology diagnosis. In the process of reaching the correct diagnosis, the case study offers a learning platform to interpret results beyond the technical findings and address pitfalls of the current techniques and CC diagnostic guidelines for achalasia, introducing an adjunctive oesophageal transit test to support diagnosis while excluding conditions that may mimic achalasia symptoms.

## Case presentation

A 20-year-old Caucasian female patient (weight 9 stones, BMI 18.7) with dysphagia and reflux symptoms was referred to our specialist clinical centre in June 2022 for an oesophageal physiology study. Prior to the referral, her gastroenterology team performed all the standard upper gastrointestinal diagnostic tests at her local general hospital and found no abnormalities to explain her symptoms. This included multiple upper endoscopies and random biopsies of the oesophagus, stomach, and duodenum to exclude oesophagitis, *Helicobacter*, and coeliac disease. Her BS study with X-ray imaging was also normal (normal contrast clearance, no mucosal thickening or abnormal anatomical features of the oesophagus and the GOJ). They assessed her symptomatic response to short courses of omeprazole, esomeprazole, and Gaviscon following the regimen recommended by the British National Formulary [38]. The patient found no clinical benefit from this. She also tried lifestyle changes, diet modification, and attended a dietitian clinic for a period. She had no other past medical history or past surgical or psychological history. Prior to the gastroenterology pathway, the patient was screened through the cancer pathway at her local general hospital, and both serological screening and radiological imaging of the torso were normal.

The primary oesophageal physiology study

**Figure 1:**
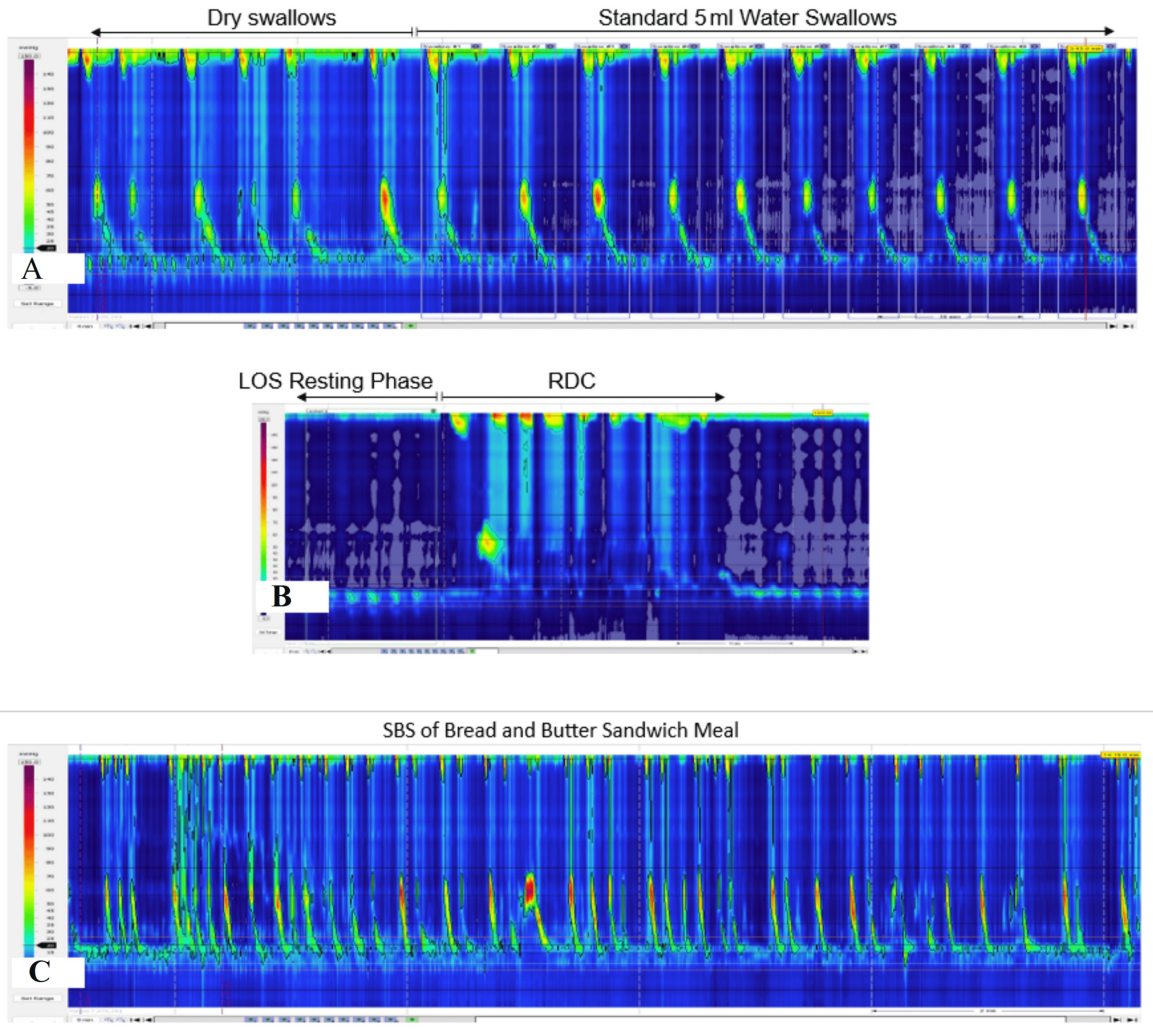
Showing IOM during the HRM study performed in the primary oesophageal physiology investigation (June 2022). (a) Peristalsis activity in the distal oesophagus during both dry swallows and the standard 5 ml water swallows; (b) GOJ resting pressure and the LOS relaxation during the RDC assessment; (c) Evidence of peristalsis during a bread and butter sandwich meal ManoView^TM^ ESO Pressure Topography Imaging with isobaric contouring at 20 mmHg magnitude (Version 3.3), Medtronic, Minneapolis, USA IOM: ineffective oesophageal motility; HRM: high-resolution manometry; LOS: lower oesophageal sphincter; RDC: rapid drinking challenge; SBS: solid bolus swallows

She successfully completed both HRM and MII-pH studies in her first appointment in June 2022, which was the primary oesophageal physiology study. The HRM study diagnosed ineffective oesophageal motility (IOM) based on CC guidelines (versions 3.0 and 4.0) [17,21]. Ten 5 ml standard water swallows invoked smooth muscle contractility of the distal oesophagus in 70% of the standard 5 ml water swallows, which were weak peristalsis (the remaining 30% of the standard 5 ml water swallows did not propagate peristalsis in the smooth muscle region of the oesophagus) (Figure 1A). The peristaltic contractility functional length was approximately 7.54 cm above the manometric GOJ at the 20 mmHg isobaric contour magnitude (the highest functional length observed was 10.6 cm). The mean DCI generated from the peristalsis was 209.4 mmHgscm (95%CI: 141.9-276.9 mmHg), and the maximum DCI observed was 414.5 mmHgscm (DCI normal range 450-8000 mmHg [17,21]).

The ManoView^TM^ software recorded and displayed the results of the CM. The overall oesophageal contractile MWA found in the oesophageal smooth muscle region was normal (54.6 mmHg, normal range 43-52 mmHg [29]). The MWA of the local oesophageal regions at 11 cm, 7 cm, and 3 cm above the GOJ were respectively 14.0 mmHg (reference range, 36-134 mmHg), 77.6 mmHg (reference range, 37-166 mmHg), and 31.7 mmHg (reference range, 41-168 mmHg [29]).

The GOJ morphology was normal without separation of the LOS band pressure from the crura diaphragm (this excluded manometric hiatus hernia in this patient). The GOJ mean pressure (18.1 mmHg) and LOS tone (8.8 mmHg) were within normal limits during the resting phase [29], and the IRP measured during the standard 5 ml water swallows was also within normal ranges (median 8.2 mmHg, mean 8.5 mmHg; 90% of the IRPs were <12.0 mmHg) [1,17,21]. Provocative testing with RDC revealed some pressurisation in the oesophageal body region with normal LOS relaxation (IRP 4.3 mmHg, normal <10 mmHg) [17] (Figure 1B). There was no peristalsis invoked on the final swallow of the RDC. On the CM screening, the LOS relaxation was 58.1% on average from LOS tone, and this was as low as 78% relaxation on RDC assessment.

SBS is performed with patients eating a butter-and-butter sandwich meal (sandwich volume 10.5x6.5x2.5 cm, with 4-5g butter spread). The patient took approximately 10 minutes to eat the sandwich (Figure 1C). In total, the patient performed 45 SBS to consume the sandwich meal. The SBS were identified by the descent of the oesophageal striated muscle contractions from the UOS in 35 SBS (77.7%). In the oesophageal smooth muscle region, 15 SBS produced peristalsis with normal DCI (33.3%), three SBS invoked fragmented peristalsis (6.7%), and 17 SBS produced weak peristalsis (37.7%). The average DCI generated by the peristalsis on SBS was 356.3 mmHgscm (95%CI, 273.8-438.8) and a maximum of 862.8 mmHgscm (DCI normal range for bread swallows: 404.60-5845.60 mmHgscm) [23]. The mean functional length of the oesophageal smooth muscle was 11.4 cm above the GOJ (95%CI: 9.54-13.3cm). The mean LOS relaxation (IRP measurement) on SBS was 12.73 (95%CI: 11.29-14.16 mmHg) (normal IRP on SBS 1.90-21.4 mmHg) [23]. On the CM screening for SBS, the overall MWA of the smooth muscle region was normal (51.1 mmHg), and the average LOS relaxation was 36.1% from tone.

In the first two minutes of eating, the patient did report a minor dysphagia symptom, but she felt she could continue eating. The dysphagia was reported on the eighth SBS, which correlated with peristalsis activity (DCI 628 mmHgscm, reference range, 404.6-5845.6 mmHgscm) and LOS relaxation (IRP 15.2 mmHg, reference range, 1.9-21.4 mmHg) [23]. The peristaltic functional length was 9.6 cm above the GOJ during this event (isobaric contour at 20 mmHg magnitude). This dysphagia symptom could not be explained by the HRM study.

In the primary HRM, we are able to compare the functional anatomy of the oesophagus during the standard 5 ml water swallows (n=10) and the SBS (n=45). In the 20 mmHg isobaric contour magnitude, a strip of HRM recording for the resting phase between swallow assessment showed higher UOS tone during the SBS than during the standard 5 ml water swallows (23.9 mmHg vs. 17.8 mmHg). The clinical significance of this is unclear, and the patient did not report oropharyngeal symptoms. The UOS residual pressure during relaxation revealed better UOS opening during the standard 5 ml water swallows (-5.74 mmHg; 95%CI, -7.91 to -3.57 mmHg) compared to the SBS (-0.148 mmHg; 95%CI, -1.31 to 1.02 mmHg) (t-statistic 4.23, p <0.001). After the swallow and the bolus passing the UOS, there was a contractile closure of the UOS, which initiated the peristalsis in the oesophageal body. The post-swallow UOS contraction from standard 5 ml water swallow (85.4 mmHg, 95%CI 80.3-90.5 mmHg) was not statistically different from SBS (84.6 mmHg, 95%CI 81.3-88.0 mmHg) (t-statistic 0.228, p=0.410). The UOS post-swallow contraction invoked peristalsis in the striated muscle of the oesophagus from the UOS. The peristaltic descending functional length from the UOS distal borderline was statistically longer on standard 5 ml water (3.22 cm; 95%CI 2.98-3.47 cm) than compared with SBS (2.91 cm; 95%CI, 2.82-3.00 cm) (t-statistic 2.91, p=0.003).

The DCI, however, of the oesophageal striated muscle was not statistically different between standard 5 ml water swallows (137.5 mmHgscm; 95%CI, 102.0-173.0 mmHgscm) and the SBS (144.1 mmHgscm; 95%CI, 103.1-185.0) (t-statistics 0.142, p=0.444). The TZ (existing between the oesophageal striated muscle and smooth muscle) was found significantly smaller on SBS (10.3 cm; 95%CI, 8.37-12.2cm) from the standard 5 ml water swallows (13.6 cm; 95%CI, 13.3-13.9 mmHg) (t-statistic 1.66, p=0.052). The oesophageal body contractility functional length of the smooth muscle region from peristalsis activity was found significantly higher on the SBS (11.4 cm; 95%CI, 9.54-13.3cm) compared to the standard 5 ml water swallows invoking peristalsis (7.54 cm; 95%CI, 5.60-9.48cm) (t-statistic 1.96, p=0.028). In addition, the smooth muscle peristaltic DCI was significantly higher for SBS (356.3 mmHgscm; 95%CI, 273.8-438.8 mmHgscm) compared with the standard 5 ml water swallows (209.4 mmHgscm; 95%CI, 141.9-276.9 mmHgscm) (t-statistics 1.68, p=0.049). The functional length of the GOJ (measured during the resting phase) was similar during the standard 5 ml water swallows and the SBS (4.4 cm vs 4.3 cm). Similarly, the GOJ mean pressure was similar during the resting phase during SBS and standard 5 mL water swallows (18.1 mmHg vs 16.5 mmHg), as was LOS tone pressure (11.0 mmHg vs 9.8 mmHg). The crura diaphragmatic contractions were more apparent on the standard 5 ml water swallows than between SBS (33 mmHg vs 28.3 mmHg). This was based on the maximum diagrammatic impingement observed during the resting phases. Finally, the LOS relaxation opening was greater during the standard 5 ml water swallows (IRP mean 8.51 mmHg, 95%CI, 7.40-9.62 mmHg) compared to the SBS (IRP mean 12.7 mmHg; 95%CI, 11.3-14.2mmHg) (t-statistics 2.74, p=0.004).

The patient successfully completed the MII-pH study with a total recording of 21 hours and 46 minutes. There were five mealtime periods recorded by the patient and a single recumbent period of 12 hours and 41 minutes for her night sleep. The 24-hour MII-pH study captured one acid reflux episode with retrograde impedance flow. This produced normal total AET (0.10%) and DeMeester score (1.0) based on the oesophageal pH capture [16]. The MNBI of the distal oesophageal mucosa was not indicative of oesophagitis (2581.70 Ω) [16,20], and the PSPW was 1 for the single acid reflux event. These findings are not predictive of pathological reflux on W-pH screening for 96 hours [36], and the patient did not undergo further reflux testing. The symptoms reported during the MII-pH study, heartburn (n=10, SI 10%, SAP 0%), epigastric pain (n=0), and regurgitation (n=0), do not show a clinically significant association with reflux events, which also excludes reflux hypersensitivity. The heartburn-reflux correlation, together with MNBI findings and the AET, would support the functional heartburn diagnosis.

The median impedance bolus clearance time was 8.0 seconds, and the longest bolus clearance time found was 9.8 seconds, which was within normal range [22,34]. The total bolus exposure time during mealtimes was 0% in the 24 hours, which was also normal [31]. The MII-pH catheter used in this study was the ComforTEC Z/pH probe (Diversatek Healthcare, Milwaukee, Wisconsin, United States), and the design was configured to reference ZAI-BG-44 (Diversatek Healthcare). The MII-pH catheter permitted capturing the gastric pH (5 cm below the manometric GOJ), and the acidic pH fraction time (pH<4) of the stomach was found to be 91.2% of the recording period.

After the primary oesophageal physiology study, her case was discussed in an MDT meeting. She underwent a chest CT scan, which was normal. She was then discharged from the gastroenterology service to her GP with the advice to prescribe prokinetic therapy for the IOM and dysphagia symptoms. She had short courses of metoclopramide, erythromycin, then famotidine, Gaviscon, and Rennie without clinical benefit. All pharmacological therapy followed the minimal to standard therapy guidelines set by the British National Formulary (Joint Formulary Committee) [38].

The second/follow-up oesophageal physiology study

**Figure 2:**
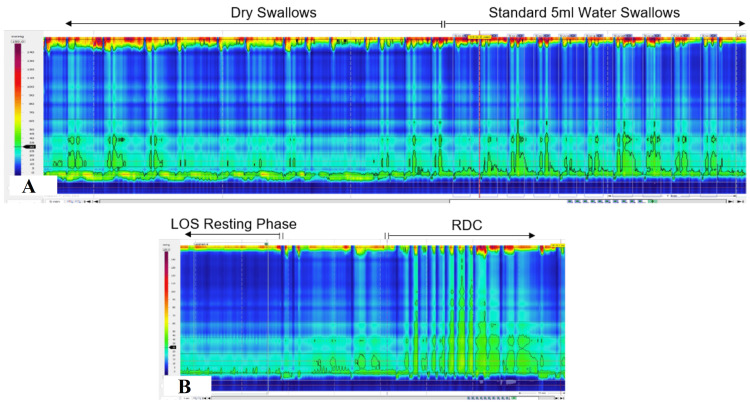
Showing absent contractility during the HRM of the second oesophageal physiology study (November 2024). (A) Showing failed peristalsis on dry swallows and on the standard 5 ml water swallows (B)Showing GOJ resting pressure phase and the non-relaxation during the RDC with pressurisation in the oesophageal body ManoView^TM^ ESO Pressure Topography Imaging at 30 mmHg isobaric contour (Version 3.3), Medtronic plc, Minneapolis, United States HRM: high-resolution manometry; LOS: lower oesophageal sphincter; RDC: rapid drinking challenge

In November 2024, the patient was referred to our specialist centre again for another oesophageal physiology investigation. On attendance, her body weight reduced to 7 stones 8 lbs (BMI 15.7), and she was mainly focused on her dysphagia symptoms in relation to her notable weight loss. She seemed distressed by the progressive dysphagia symptom and daily postprandial regurgitation of undigested bolus. She felt her continual weight loss was beginning to impact her work duties and her quality of life as a young adult. The HRM of the second oesophageal physiology study was successfully completed, and data revealed failed peristalsis in the oesophageal body (100%) and elevated IRPs for the LOS relaxation during the 5 ml standard water swallows (IRP median 30.6 mmHg, mean 31.2 mmHg). The dry swallows captured also showed similar patterns of the motility disorder. The HRM in the second oesophageal physiology study was diagnostic of achalasia (subtype I) based on the CC guidelines [17,21,24] (Figure 2A). The RDC demonstrated significant panoesophageal pressurisation in the oesophageal body from fluid retention and a poor GOJ opening (IRP 24.7 mmHg), which also supports the achalasia finding (Figure 2B). The patient adopted a lifestyle of not eating bread/starchy food since her first oesophageal physiology test and declined the bread-and-butter sandwich meal during the second oesophageal physiology study.

**Figure 3:**
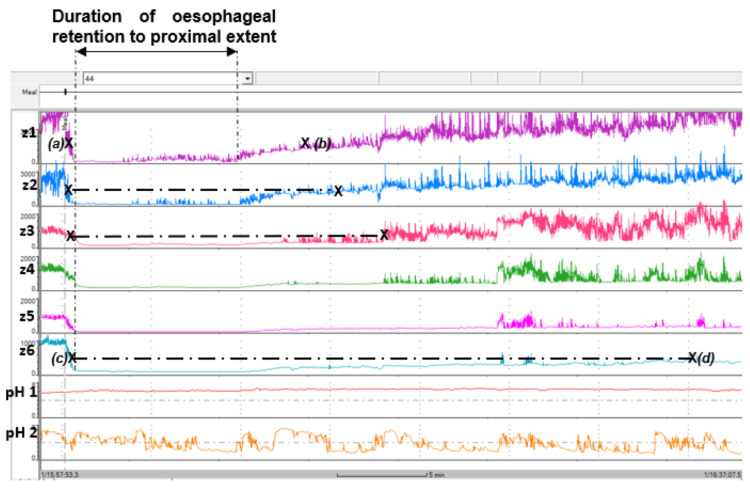
Showing alternating current changes on impedance recording between oesophageal mucosa and saline during the impedance transit test. This study was performed during the MII-pH study of the second oesophageal physiology study on November 2024. The MII-pH catheter design was as follows: -Impedance sensors z1, z2, z3, z4, z5, and z6 were respectively positioned 17cm, 15cm, 9cm, 7cm, 5cm, and 3cm above the manometric GOJ. -pH sensors 1 and 2 were respectively positioned 5cm above the manometric GOJ (distal oesophagus) and 5cm below the manometric GOJ (stomach). The first ‘X’ marking on the impedance traces at each sensor location on the horizontal plane is indicated at the 50% impedance drop for the saline entering the oesophageal segment, and the second ‘X’ marking on the same horizontal plane is indicated at 50% impedance recovery from the saline clearance from the oesophageal segment. The time duration between the two ‘X’ markings on each horizontal plane is the transit time at each location of the oesophagus. The saline transit at the location of impedance sensors z2, z3, and z6 corresponds to the oesophageal transit in the proximal, mid, and distal oesophagus. Saline transit between sensors was also computed. Points (a) to (c) measure the intraoesophageal transit from proximal to distal oesophagus from the first swallow of saline. Points (b) to (d) measure the oesophageal clearance time from the proximal to distal oesophagus from the final swallow of saline; Points (a) to (d) measure the total saline transit time of the oesophagus. BioVIEW Analysis software version 5.7.1.0 (Diversatek Healthcare, Milwaukee, Wisconsin, United States) MII-pH: multichannel impedance-pH; GOJ: gastro-oesophageal junction

The MII-pH catheter in the second oesophageal physiology study was successfully intubated, but the catheterisation could only be tolerated for slightly more than one hour. The patient returned to the laboratory after attempting to eat her lunch meal with the catheter in situ. At the start of the MII-pH study, an impedance transit test was performed, for which the patient managed to drink 150 ml of saline [5]. The saline transit was clearly identifiable by the changes in alternating current between oesophageal mucosa and saline in the oesophagus [4,5,9,22,28,31,34] (Figure 3). The average oesophageal mucosal impedance prior to swallowing the saline was 1844.40 Ω, which reduced to 199.0 Ω on average when the saline drinking phase started.

The saline transit at each impedance sensor location was measured from the 50% impedance drop by the saline presence to the 50% impedance recovery from the saline clearance (Figure 3) [22]. There were approximately nine minutes when all the impedance sensors were measuring saline impedance, which suggests retention of the full length of the oesophagus. The saline clearance at the sites of the impedance sensors above the GOJ (z1 (17cm), z2 (15cm), z3 (9cm), z4 (7cm), z5 (5cm) and z6 (3cm)) were respectively 12.9 minutes, 14.7 minutes, 17.4 minutes, 23.2 minutes, 33.1 minutes, and 33.7 minutes. BS study for achalasia has been correlated to oesophageal impedance clearance of saline from the proximal oesophagus (z2, quartile range for achalasia is 0.51-22 minutes), mid oesophagus (z3, quartile range for achalasia is 1.20-54.5 minutes), and distal oesophagus (z6, quartile range for achalasia is 4.0-60 minutes) [5]. Comparing the saline transit in the patient case from the proximal oesophagus (z2, 14.7 minutes), mid oesophagus (z3, 17.4 minutes), and distal oesophagus (z6, 33.7 minutes) would predict achalasia [5]. The first swallow of saline transit from z1 to z6 was 7.20 seconds in duration (Figure 3, points between ‘a' and ‘c’). The final swallow for oesophageal clearance duration was 20.4 minutes (see mucosal impedance recovery from z1 to z6 at points ‘b’ and ‘d’). The total saline transit time measured from the proximal to distal oesophagus was approximately 33.9 minutes (see duration from point ‘a’ to ‘d’). The oesophageal volumetric discharge rate for the oesophageal impedance transit test was found to be 4.42 mL/minute.

Oesophageal physiology primary and follow-up study comparison

There was a 30-month gap period between the primary and the follow-up HRM studies, for which the progression of achalasia (subtype I) from IOM was observed. In this clinical case, the weight loss/BMI reduced by approximately 16.0% in this period (Table 1). This reduction equates to achalasia development being associated with a weight loss of 8.4 pounds per year or a BMI reduction of 1.2 per year. The patient’s BMI went from a healthy weight range to clinically underweight, which would increase her risks of malnutrition, weakened immunity, and developing issues with bone and reproductive health. The manifestation of achalasia was from loss of inhibitory innervation of the LOS and loss of peristaltic contractility in the oesophageal body. This development has interestingly altered the abdominal-thorax pressure dynamics. In the resting phase, the mean abdominal pressure (-2.30 mmHg) was found to be higher than the mean thorax pressures in the distal (-4.8 mmHg), mid (-5.0 mmHg), and upper (-4.1 mmHg) chest regions during the primary HRM study when IOM was diagnosed. This pressure dynamics between the stomach and oesophagus would behave as a vacuum effect, which supports the theory for bolus regurgitation, rumination, and reflux to occur. The development of achalasia completely transformed the pressure dynamics, which captured the opposite of the primary HRM study. The abdominal mean pressures (1.40 mmHg) were significantly lower than the thorax pressures of the distal (18.6 mmHg), mid (14.7 mmHg), and upper (8.60 mmHg) chest regions.

**Table 1:** Showing clinical measurements recorded during resting phase or on standard 5 ml water swallows during HRM in June 2022 (primary HRM study) and November 2024 (followed-up HRM study). NOTE: Additional testing (adjunctive) has been indicated by MWS, RDC or SBS. UOS: upper oesophageal sphincter; DCI: distal contractile integral; TZ: transition zone; GOJ: gastro-oesophageal junction; LOS: lower oesophageal sphincter; IRP: integrated relaxation pressurisation; MWS: multiple water swallows; RDC: rapid water drinking challenge; SBS: solid bolus swallow; HRM: high-resolution manometry; CM: conventional manometry

Visitation date (month/year)	June 2022	November 2024	Normal Range/Notes
Patient age at visit (years)	22 years & 6 months	24 years & 11 months	
Weight	9 st 10 lbs	7 st 8 lbs	Ideal body weight 9 st 4 Ibs to 12 st 2 lbs
BMI (kg/m^2^)	18.7	15.7	18.5–24.9 kg/m^2^
Abdominal/Thoracic Pressure Dynamics			
Abdominal pressure (mmHg), mean (range)	-2.30 (-5.6–0.8)	1.40 (-2.5 to 12.2)	Pressure profile taken of 5 cm strip below the GOJ (no established normal range)
Distal thoracic pressure (mmHg), mean (range)	-4.8 (-10.8–3.4)	18.6 (11.4–24.7)	Pressure profile of 5 cm strip above the GOJ (no established normal range)
Mid thoracic pressure (mmHg), mean (range)	-5.0 (-10.5–0.3)	14.7 (5.8–24.7)	Pressure profile of 5 cm strip between 5 cm and 10 cm above the GOJ (no established normal range)
Upper thoracic pressure (mmHg), mean (range)	-4.1 (-9.7–1.9)	8.60 (2.90–15.9)	Pressure profile of 5 cm strip between 10 cm and 15 cm above the GOJ (no established normal range)
Upper Oesophageal Sphincter			
Functional length (cm)			No recording of the proximal borderline of the UOS
Tone/Resting pressure (mmHg)	17.8	71.6	Normal 34–104mmHg
Relaxation (residual pressure) (mmHg), mean (range)	-5.74 (-7.91 to -3.57)	4.29 (3.30–5.28)	<12.0mmHg
Relaxation duration (msec), mean (range)	730 (700.4–759.6)	816 (783.8–848.2)	74–365 msec
Post-swallow contractile pressure (mmHg), mean (range)	85.4 (80.31–90.5)	118.9 [98.0–139.8]	No established normal range
Oesophageal Body Motility			
Standard 5ml Water Swallow (n=10)			
Striated muscle functional length (cm), mean (range)	3.22 (2.98–3.47)	0.90 (0.81–0.99)	No established normal range
Striated muscle DCI (mmHgscm), mean (range)	137.5 (102–173)	35.6 (24.3–46.9)	No established normal range
Average TZ size (cm), mean (range)	13.6 (13.3–13.9)	28.3 (28.2–28.5)	Normal <2cm [17,21], <3cm [24]
Smooth muscle functional length (cm), mean (range)	7.54 (5.60-9.48)	0	No established normal range
Peristalsis, n (%)	7 (70%)	0 (0%)	Normal >50% [21,24], >40% [17]
Failed peristalsis, n (%)	3 (30%)	10 (100%)	Normal <50% [17,21,24]
Weak peristalsis, n (%)	7 (70%)	0 (0%)	Normal <50% [21,24], <40% [17]
Fragmented peristalsis, n (%)	0 (0%)	0 (0%)	Normal <50% [21, 24] <70% [17]
Normal peristalsis, n (%)	0 (0%)	0 (0%)	Normal >40% [21, 24], ≥40% [17]
Peristaltic DCI (mmHgscm), mean (range)	209.4 (141.9–276.9)	0	Normal 450 – 8000 [17,21,24]
Peristaltic DCI maximum (mmHgscm)	414.5	0	Normal 450 – 8000 [17,21,24]
GOJ Morphology & Physiology			
Resting Phase Assessment			
Manometric hiatus hernia	No	No	Normal No
Functional length (cm)	3.2	2.9	No established normal range
Trans gradient GOJ pressures (mmHg), mean (range)	8.6 (-7.5 to 40.9)	22.8 (3.9–47.4)	No established normal range
GOJ mean pressure (mmHg)	18.1	27.9	Normal 13–43mmHg [29]
LOS tone pressure (mmHg)	8.8	23.7	Normal 4.8–32.0mmHg [29]
IRP (standard water swallows) (mmHg), mean (range)	8.51 (7.40–9.62)	31.2 (29.4–33.0)	Normal <15mmHg [21,24], <12mmHg [17]
Normal relaxations, n (%)	9 (90%)	0 (0%)	Normal >50% having normal relaxation
Adjunctive Testing (liquids)			
Peristaltic DCI on MWS	Not performed	Not performed	
IRP on RDC (mmHg)	4.30	24.7	Normal <10mmHg [17]
Oesophageal impedance transit	Solid bolus clearance rate (mean 8.0 seconds, maximum 9.8 seconds)	Liquid clearance rate (20.4 minutes). Total liquid transit time for 150mL (33.9 minutes).	No established normal range
Adjunctive Testing (SBS)			
UOS Physiology			
Resting pressure (mmHg)			
Relaxation (residual pressure) (mmHg), mean (range)	-0.148 (-1.31 to 1.02)		
Post-swallow contractile pressure (mmHg), mean (range)	84.6 (81.3–87.9)	SBS was not performed	
Oesophageal Body Motility			
Striated muscle functional length (cm), mean (range)	2.91 (2.82–3.00)		
Striated muscle DCI (mmHgscm), mean (range)	144.1 (103.1–185.0)		
TZ length (cm), mean (range)	10.3 (8.37–12.2)		
Smooth muscle functional length (cm), mean (range)	11.4 (9.55–13.3)		
Peristalsis, n (%)	35 (77.7%)		
Failed peristalsis, n (%)	10 (22.2%)		
Weak peristalsis (%, n)	17 (37.7%)		
Fragmented peristalsis, n (%)	3 (6.7%)		
Normal peristalsis, n (%)	15 (33.3%)		
Peristaltic DCI mean (mmHgscm), mean (range)	356.3 (273.8–438.8)		
Conventional Manometry			
Standard 5ml water swallows (n=10)			
Overall MWA, mean (range)	54.6 (40.5–68.8)	12.7 (10.1–15.3)	Normal 43–152mmHg [29]
MWA at 11cm above GOJ, mean (range)	14.0 (11.9–16.1)	10.8 (8.30–13.3)	Normal 36–134mmHg [29]
MWA at 7cm above GOJ, mean (range)	77.6 (55.1–100)	13.3 (10.6–16.0)	Normal 37–166mmHg [29]
MWA at 3cm above GOJ, mean (range)	31.7 (24.0–39.3)	12.0 (9.53–14.5)	Normal 41–168mmHg [29]
LOS relaxation (%), mean (range)	58.1 (49.8–66.4)	11.8 (6.90–16.7)	Normal >40.0%
SBS (n=45)			
Overall MWA, mean (range)	46.4 (37.0–55.8)		
MWA at 11cm above GOJ, mean (range)	13.5 (11.3–15.8)	SBS was not performed	
MWA at 7cm above GOJ, mean (range)	54.6 (44.2–65.0)		
MWA at 3cm above GOJ, mean (range)	38.2 (29.1–47.2)		
LOS relaxation (%), mean (range)	36.1 (28.9–43.4)		
HRM Diagnosis	IOM	Achalasia (subtype I)	
CM Diagnosis	Normal	Achalasia	

The upper oesophagus, which is composed primarily of striated muscle, unexpectedly also revealed changes in the physiology by the achalasia development, which was captured on HRM studies. The maximum UOS tonicity captured during the resting phase seems to be much higher in the follow-up HRM study when achalasia was diagnosed (71.6 mmHg vs 17.8 mmHg), and the residual pressures captured during the standard 5 ml water swallows were also statistically higher from the development of achalasia (4.29 mmHg vs -5.74 mmHg, t-statistic 9.52, p<0.001). This would result in poorer UOS opening due to the achalasia development. Another interesting phenomenon observed by the achalasia development was the relaxation duration, which was statistically longer in the achalasia development (816 ms vs 730 ms, t-statistic 4.42, p<0.001).

We examined the UOS post-swallowing contractile closure for the standard 5 ml water swallows, which was found to be higher in the achalasia state (118.9 mmHg vs 85.4 mmHg, t-statistic 3.52, p=0.001). This UOS post-swallow contractility descent of the striated muscle from the UOS distal borderline significantly reduced in the functional length when achalasia developed (0.90 cm vs 3.22 cm, t-statistic 20.1, p<0.001). This reduction in the striated muscle functional length was by a factor of 3.58, and there was a similar reduction in the striated muscle DCI in the achalasia state (137.5 mmHgscm vs 35.6 mmHgscm, t-statistic 6.58, p<0.001). As expected in the achalasia development, no smooth muscle activity in the oesophagus and therefore follow-up HRM study revealed longer TZ/peristaltic break from the striated muscle (28.3 cm vs 13.6 cm, t-statistics 87.7, p<0.001), no measurable smooth muscle functional contractility length (0 cm vs 7.54 cm, t-statistics 8.78, p<0.001) or DCI vigour (0 mmHgscm vs 209.4 mmHgscm, t-statistic 7.01, p<0.001), and no peristalsis activity (0% vs. 70%, χ2=10.77, p<0.001). The oesophageal body functional length between the two sphincters was greater in the achalasia development (29.5 cm vs 25.5 cm), which may be due to reduced oesophageal tissue tonicity/absence of contractility.

From the GOJ morphology, there was no development of hiatus hernia on HRM study in the progression of achalasia, and the GOJ functional length was similar (2.9 cm vs 3.2 cm). The progression to achalasia showed higher transgradient GOJ pressure (22.8 mmHg vs 8.6 mmHg), GOJ mean pressure (27.9 mmHg vs 18.1 mmHg), and LOS tone (23.7 mmHg vs 8.8 mmHg) during the resting phase of the studies (paired t-test of anti-reflux barrier pressure changes: t-statistic 8.12, p=0.015). The development of achalasia significantly increased IRP on standard 5 ml water swallows (31.2 mmHg vs 8.51 mmHg, t-statistics 24.4, p<0.001) and prevalence of normal IRPs (<12.0 mmHg) on standard 5 ml water reduced from 90% to 0% (χ2=10.36, p<0.001).

In the MII-pH study of the primary oesophageal physiology study, the total bolus transit of SBS was measured during the mealtimes. This was measured from antegrade impedance flow from each SBS from sensors z1 to z6 [4,31]. The average bolus transit of SBS was 8.0 seconds, and the longest was 9.8 seconds. The literature has documented that bolus transit solids and semi-solids take longer transit times than liquids in general [28]. However, the oesophageal transit of liquid was found to be longer in this clinical case, which would support the diagnosis of achalasia. When achalasia was developed based on HRM screening, the oesophageal impedance transit on the first swallow of saline took 7.20 seconds between sensors z1 to z6, which would be considered normal [22,34]. However, there was no passage of saline into the stomach but rather evidence of saline filling the oesophagus from the impedance changes from mucosa to saline at each sensor for a prolonged duration (as previously discussed). The oesophageal clearance time after retention took 20.4 minutes to clear the oesophagus, and the total saline transit time was 33.9 minutes. Oesophageal impedance transit duration would be consistent with achalasia [5].

The oesophageal motility captured on the CM in the follow-up study revealed notably reduced contraction amplitudes, which were simultaneous in nature and found in the smooth muscle region of the oesophagus. The overall MWA was statistically lower in the achalasia development (12.7 mmHg vs 54.6 mmHg, t-statistic 13.02, p<0.001). As was the local regions at 11 cm above GOJ (10.8 mmHg vs 14.0 mmHg, t-statistic 1.73, p=0.054), 7 cm above the GOJ (13.3 mmHg vs 77.6 mmHg, t-statistic 13.9, p<0.001), and 3 cm above the GOJ (12.0 mmHg vs 31.7 mmHg, t-statistic 8.59, p<0.001). The LOS relaxation (%) on CM was normal in the primary manometry study (58.1%), and poor relaxation was observed in the follow-up manometry study when achalasia developed (11.8%) (t-statistic 10.9, p<0.001). The CM is also consistent with achalasia in the follow-up study. The patient did not undergo SBS in the follow-up manometry study to compare with SBS from the primary manometry study.

## Discussion

The prevalence of achalasia is approximately found in 10 people for every 100,000, which does not seem to predominantly affect a particular age, gender, or race [39,40]. The annual incidence rate of achalasia was stable for 50 years (approximating 0.5 cases for 100,000 population) [39] until the advent of HRM and the CC guidelines [24]. The significant increase in the achalasia diagnosis with CM and HRM technologies can be compared in more recent years, which revealed eight per 100,000 in 1997 and 15 per 100,000 in 2017 [40]. This is an increase of 87.5% in the diagnostic yield from the technological advancements [24]. Other research practitioners have also observed this increase in their practice [24,26], and we also advocate the HRM practice over CM.

We kindly remind oesophageal physiology practitioners and centres that the clinical technology in manometry has reached saturation in the last two decades and only refinements in the diagnostic classification have been undertaken [17,21,24]. The diagnosis for achalasia has been consistent throughout the CC editions [17,21], and the first decade of the HRM in clinical practice has unveiled technical advantages over CM, which include a user-friendly application and quicker to perform high-quality oesophageal motility studies [26]. HRM has produced standardisation and objectivity in the clinical diagnosis across centres in the Western world [17,21,24]. Clinical educators found their trainees to be more proficient in their HRM learning, including investigative performance, demonstrating more confidence and accuracy for making diagnoses [41]. General physicians also seem to better understand HRM recording over the CM recording [41]. There are no classification or guidelines that parallels the CM classification (Spechler and Castell Classification) to HRM (CC), and the two do not synergise as they were developed approximately a decade apart. Practitioners and centres continuing with CM would mean dumbing down in the technological advancements and not having closely packed sensors, which would mean the inability to measure the spatial relationship in oesophageal body function (i.e., isolation of oesophageal striated and smooth muscles, CDP) [7,8,10,24]. In this case study, the HRM in the primary study demonstrated IOM, but overall MWA of the oesophageal muscle region was normal. This would be considered a normal CM study. This case study supports the findings of a randomised study that compared outcomes of HRM and CM for detecting achalasia [26].

Achalasia is a rare condition, and manometry is the gold-standard diagnostic tool to diagnose achalasia. In the technological advancements, HRM seems to supersede CM in the detection of achalasia. This is paramount so patients can receive the correct treatment in a timely fashion and prevent worsening symptoms or developing other conditions. The management of achalasia is mostly undertaken in outpatient settings, but more recently, achalasia patients are increasingly requiring more hospitalisations per year [40]. This may be due to achalasia being more commonly diagnosed in young and middle-aged adults [40], who may opt for surgical management [27]. There is no cure for achalasia, and clinical management is palliative for the symptoms (dysphagia, odynophagia, undigested bolus regurgitation, weight loss) [24,30,39,42] and prevention of dilated oesophagus or megaoesophagus developing [42]. The successful clinical management of achalasia can be correlated to the trends in the reduction of death rates per annum in patients with achalasia as their principal diagnosis [40]. The death rate as the principal cause of achalasia was reported as 1.98%, and the survival rate with achalasia is no different from the general patient population. Patients with achalasia generally require invasive therapies that are repeated to reduce the LOS tonicity, thereby easing bolus transport to the stomach. This form of management is associated with reflux oesophagitis, which is a complication found in up to 19.7% of achalasia patients [42].

The pathology of achalasia is the loss of inhibitory nitrinergic neurons in the oesophageal myenteric plexus that manifests in the dysfunction of the lower oesophagus and GOJ, which is captured in oesophageal physiology practice. The aetiology is unknown, but hypotheses include disease of the extrinsic (vagal) nervous system, inflammation within the oesophageal myenteric plexus, genetic inheritance, autoimmune cause, and previous viral infection. The diagnosis of achalasia in clinical practice is generally from an idiopathic origin. The diagnosis is initially captured by the BS study, from capturing achalasia features (contrast retention, anatomical abnormality of GOJ/lower oesophagus), which can also exclude pseudoachalasia. Achalasia is then confirmed by a manometry study, which captures aperistalsis and non-relaxation of the LOS [17,21,24]. In the patient’s case, there were no features of achalasia on the BS study or on chest CT because imaging was performed during the pre-achalasia state (it can be noted that radiological investigations could not identify the pre-achalasia). Oesophageal physiology testing with impedance sensors has the potential to measure oesophageal transit [4,5,9,28,31], but this at its infancy. Some recent data have shown oesophageal impedance transit to be more sensitive than the BS study to explain dysphagia from the oesophageal clearance rate [5], and HRM has excluded BS-diagnosed achalasia in 16.0% cases from capturing evidence of peristalsis [5]. The concordance of oesophageal impedance transit to HRM for achalasia is superior to the concordance observed for the BS study and HRM for achalasia [5].

The statistical prevalence of achalasia is only based on confirmed cases from manometry diagnosis. However, achalasia may go undetected if the manometric study was performed at the pre-achalasia state. In the clinical case, the HRM diagnosis of IOM and the CM of normal study were found (see Table 2). Furthermore, the IOM found on HRM during the primary oesophageal physiology study was compatible with the patient’s main complaint at the time (reflux symptoms). As the patient had reflux symptoms and was not responding to anti-reflux medication, the primary oesophageal physiology study was actually conducted to consider for anti-reflux surgery. The MII-pH study in the primary study excluded pathological reflux, and the notion of anti-reflux surgery was abandoned. The IOM was presented (unknown state of pre-achalasia), and the patient was complaining of reflux symptoms. This would suggest the patient was having functional heartburn, and that would completely set the patient’s treatment pathway into a different trajectory (i.e., involving neuromodulators and psychotherapy). But the IOM diagnosis on HRM is an oesophageal abnormality that halted this clinical pathway. The CM study being normal in this clinical case may not have the same outcome. The literature has documented achalasia patients having heartburn without having pathological reflux on oesophageal pH screening [39]. The reflux symptoms in achalasia (or pre-achalasia state) may be caused by residual bolus retention of acidic bolus or lactic acid fermentation of prolonged bolus retention in some foods. This would not be captured on MII-pH recording as retrograde impedance flow as observed in gastric reflux events [16,20,22,31,34], and may be undetected if the bolus resides between the sensors (i.e., impedance or pH sensors) and not on the sensors (the sensors are positioned sparsely within the oesophagus). More importantly, IOM in general has not been extensively studied with oesophageal transit, and the oesophageal transit in this case study was abnormal when the achalasia developed.

We were only able to deduce the IOM as the pre-achalasia state retrospectively because the second oesophageal physiology study confirmed the achalasia. So a valid line of questioning may be what prompted the second oesophageal physiology study, given that gastroenterology patients do not normally have repeat oesophageal physiology studies, especially in the case of a patient who is cared for at a general hospital (there are also no clinical guidelines or recommendations in the literature for repeating the oesophageal physiology or having oesophageal physiology surveillance). We wondered if the achalasia prevalence would increase more in patients complaining of progressive dysphagia and weight loss with IOM diagnosis in the absence of pathological reflux, and develop achalasia on a follow-up oesophageal physiology study. This was the patient's situation, and it was also the patient’s perseverance to be referred to have a second oesophageal physiology study as her main symptom shifted from dyspepsia to dysphagia, and the dysphagia became more apparent to her with the unintended notable weight loss and fatigue.

The patient informed us in her second visit that she undertook an extensive internet/Google search of her symptoms (dysphagia, bolus regurgitation, and weight loss), and she encouraged her physician to refer her for a follow-up oesophageal physiology study. From the patient’s perspective, the primary HRM was the only clinical test that found something abnormal in her oesophagus after undertaking countless investigations. She wanted to pursue this line of investigation in light of the progressive dysphagia and weight loss. We had tremendous empathy for this young lady, having gone through numerous medical specialties, repeated clinical tests, being discharged, and undertaking her own research for her health condition. We also reminded her of the growing criticism of the quality of healthcare information found on the internet [43]. We would like to remind the readers that more than 70,000 websites disseminate health information on the internet, which are accessed by >50 million young people who are trying to find information about their health condition [43]. This may have consequences for the modern healthcare system as found in this patient who was determined to uncover her health condition and requested repeating these specialised clinical tests. The patient works in finance and does not have higher education in health or bioscience.

Retrospective review of the primary oesophageal physiology study revealed insights of the features to suspect the development of achalasia. In parallel, the latest technology in oesophageal physiology (HRM and MII-pH) used in the clinical case was also able to exclude rumination syndrome, which has similar symptoms to achalasia. The continuing use of the CM system over HRM will reduce the chances of capturing dysmotility [24], which also cannot distinguish between achalasia and rumination. Rumination was excluded in the patient case on HRM, from no evidence of intragastric pressurisation observed during the solid bolus meal or in the postprandial phase. The close packing of pressure sensors measured a 5 cm strip of the gastric pressure (below manometric GOJ), which permitted this assessment. The patient, in addition, did not report postprandial regurgitation or vomiting after her sandwich meal. Likewise, the MII-pH study recording during the primary oesophageal physiology study also did not record the typical repetitive cyclic vomiting/regurgitation symptoms clustering in the postprandial phase periods. All five mealtimes during the MII-pH study did not record the gastric pH patterns for rumination syndrome. This would typically be captured and displayed by neutral gastric pH postprandially, with frequent non-acid reflux events and regurgitation symptoms occurring. The non-acid reflux and regurgitation symptoms would ease as gastric pH tends towards ≤4. This is when the partially digested bolus in the stomach is no longer palatable, and a fresh meal is consumed, and the cycle of rumination postprandially is observed and synchronised to the gastric pH profile. The gastric pH buffering from acidic pH was in 8.8% of the recording time and localised to mealtimes only. The acidic pH (<4) found to be 91.2% of the recording period would also exclude achlorhydria and atrophic gastritis. The single sensor C-pH and W-pH technologies can only measure the distal oesophageal pH and cannot investigate the gastric pH.

From comparing the two oesophageal physiology studies, there is evidence of the pre-achalasia manifesting as a minor motility disorder (IOM), which developed into a severe form of achalasia (subtype I) in the 30 months. The topography contour plots of the peristalsis activity in the IOM were approximately 7.5 cm (95%CI: 5.6-9.5 cm) above the manometric GOJ with the TZ of approximately 13.6 cm (95%CI: 13.5-13.9 cm) on standard 5 ml water swallows (Figure 1A). The distal oesophageal peristalsis was not apparent on the HRM follow-up study, and instead, intrabolus pressurisation was observed above the GOJ during the dry swallows and standard 5 ml water swallows at 30 mmHg isobaric (Figures 2A). The intrabolus pressurisation did capture a small increment in the contractile MWA (10.1-15.3 mmHg) throughout the oesophageal smooth muscle region (Table 1). This should not be confused with peristaltic contractions or remnants of peristalsis at low amplitudes on CM.

In the primary HRM study, the peristaltic activity in the IOM was localised in the distal oesophagus with a mean oesophageal DL of 4.6 seconds (the DL cannot be measured on CM as the CDP would not be identified from the reduced pressure sensors). This degree of oesophageal DL on HRM is actually borderline to diagnosing oesophageal spasm (spasm is diagnosed when the distal oesophageal latency is <4.2 seconds) [1,17,21]. This finding may indicate that the oesophageal body motility was at a transitional phase. This is of clinical interest as the oesophageal spasm development is a pathophysiology found in the early stages of achalasia (subtype III) [17,21,24]. The progression of achalasia is then into subtype II with panoesophageal pressurisation feature (or simultaneous contractions) in the oesophageal body, which would be observed during the standard 5 ml water swallows [17,21,24]. The deterioration of the simultaneous contractions leads to subtype I achalasia, which was found in the HRM follow-up study after 30 months. The borderline finding of oesophageal spasm in the oesophageal body should warrant repeating the oesophageal physiology study between 12 to 18 months to investigate the development of oesophageal spasm. In terms of the oesophageal physiology surveillance, it is important to note that the development of achalasia (subtype I) from IOM occurred in 30 months. Thus, the development at stages of subtypes III and II of achalasia was missed in this timeframe, which may have treatment implications [30]. Therefore, it is reasonable to suggest that the oesophageal physiology surveillance for suspected achalasia be performed annually, so that the mild form of achalasia (subtype II) condition could be captured and treated at the appropriate timescales. The oesophageal body pattern defines the subtype of achalasia, which has treatment implications (CM testing cannot subtype the achalasia stage/progression). In the literature, subtype II achalasia has the most effective treatment response to dysphagia symptoms and for longer periods, whether treating with balloon dilatation or surgical myotomy [30,39].

For young patients, such as in the clinical case study, surgical myotomy treatment outcome for achalasia seems to supersede balloon dilatation in the symptomatic response (86.7% vs 78.0%), which is based on a review of 54 studies comparing 1,487 patients who underwent myotomy and 1,144 patients who underwent balloon dilatation [39]. This is consistent with Eckardt and colleagues [27], who found balloon dilatation to be associated with higher recurrence of achalasia at shorter follow-up. The young age also seems to be an unfavourable factor post dilatation for (i) symptom recurrence, (ii) re-occurring elevated LOS pressure, and (iii) developing a dilated oesophagus. If the surgical option was not available, the success rates of balloon dilatation at a two-year follow-up review revealed the longest for subtype II achalasia (96%), which was followed by subtype I achalasia (81%) and subtype III achalasia (66%) [30]. Repeated balloon dilatation may result in the development of a weak anti-reflux barrier [27], and reflux oesophagitis has been reported as a complication from repeated therapies in up to 19.7% of achalasia cases [42]. Therefore, patients may still require surgical intervention for the risk of oesophagitis (i.e., myotomy with fundoplication wrap). The key seems to be targeting the treatment during the achalasia subtypes III and II staging, which is reflected in appropriately capturing the achalasia progression on the oesophageal physiology follow-up study. Oesophageal physiology follow-up studies or surveillance in the post-treatment phase could also be useful to predict long-term success of the achalasia treatment [27], which is also not in clinical practice or set in the diagnostic guidelines.

Another major finding in the primary HRM study that may clinically justify repeating the oesophageal physiology is from the quantitative measurements obtained of the LOS muscle function. During the HRM of the primary oesophageal physiology study, the IRP measurements obtained during the standard 5 ml water swallows were normotensive or within an adequate degree of relaxation (IRP median 8.2 mmHg, mean 8.5 mmHg) based on the technical threshold outlined by the CC guidelines [17,21,24]. However, this relaxation threshold, despite being within normal range, was merely an overall reduction by 6.82% (median difference) or 3.41% (mean difference) from the LOS tone pressure (8.8 mmHg). This is negligible pressure changes during the standard 5 ml water swallows and can be interpreted as non-relaxation of the LOS. This is a pitfall in the CC guidelines that is unable to detect a degree of GOJ obstruction that occurs below the 12 mmHg threshold. This is because the CC guidelines focus on the LOS relaxation threshold irrespective of the LOS tone [17,21,24]. The future edition of the guideline may wish to incorporate the extent of the relaxation pressure (IRP) from the normotensive LOS tone pressure. This will permit the detection of low-grade GOJ outflow obstruction occurring with normotensive IRP. The MII-pH study from the primary oesophageal physiology study also supports the theory of the GOJ outflow obstruction occurring with normotensive IRP. This is from observing incredibly reduced oesophageal acid exposure. The number of acid reflux episodes captured was 1, while the normal range is up to 40 episodes per 24 hours [16,22], and up to 80 is still regarded as inconclusive [16]. The total AET found was 0% while normal is up to 6.0% of the 24-hour MII-pH study [16]. The incredibly reduced oesophageal acid reflux exposure could be a marker of the non-relaxing LOS. In contrast, the CM captured the LOS relaxations, on the 10 standard 5 ml water swallows, was significantly higher compared to HRM capture of the same 10 standard 5 ml water swallows during the primary HRM study (58.1% vs 3.41%, t-statistic 10.2, p<0.001). Unlike the HRM, CM captured an acceptable degree of relaxation on the 5 ml standard water swallows in the pre-achalasia state (normal LOS relaxation >40% on CM Sierra Scientific Instruments) [29], which is a poor indicator of achalasia developing.

In the primary HRM study, the RDC revealed the LOS relaxation pressure to be approximately reduced by 50% from the LOS tone (4.3 mmHg) with some degree of intrabolus pressurisation in the oesophageal body. We received the RDC assessment on CM screening and found the LOS relaxation by 78% (or the lowest residual pressure 1.94 mmHg), and the intrabolus pressurisation could be confused with multiple contractions. The intrabolus pressurisation in the oesophageal body on HRM raises suspicion of temporary fluid retention before the fluid weight assisted the traversing through the GOJ and into the stomach (the patient was seated in the upright position during the swallow tests). In the follow-up HRM study, the RDC showed poor LOS relaxation (IRP 24.7 mmHg, normal <10 mmHg) [17] with higher intrabolus pressurisation developing above the manometric GOJ from the retention (Figure 2B). This pressurisation was also notable at 30 mmHg isobaric contour on Figure 2B. This finding was supported by the oesophageal impedance transit test, which was performed during the MII-pH study in the follow-up oesophageal physiology study. The oesophageal impedance transit technique used is relatively novel but simple to conduct [5]. The method permits oesophageal physiology practitioners to measure the transit of the oesophagus [5] in parallel to the motility during a single oesophageal physiology clinic. The combination of the two provides a comprehensive assessment of the oesophageal physiology that can fulfil the latest Chicago Classification guideline (version 4.0) [17]. Thus, patients may not require follow-up referrals for a BS study in unequivocal findings of HRM or confirmation of major motility disorders [17]. Oesophageal impedance transit was possible from the significant change in impedance from the oesophageal mucosa and saline during the retention phase. In this clinical case, the impedance reduced by 87.3%, which allowed easy interpretation of the oesophageal transit. The patient was only able to drink 150 ml of saline solution of the 200 ml for the oesophageal impedance transit study. We did not impose the patient to drink the full 200 mL in case of the patient having a regurgitation or vomiting episode. Regardless of reduced intake of saline, oesophageal clearance of the saline was poor, which explained the dysphagia symptom from retention and the achalasia [5]. The distal oesophageal transit time was 33.7 minutes in this patient's case. Clinical dysphagia from retention is found when the distal oesophageal transit exceeds 47.4 seconds, and the distal oesophageal transit exceeding 5.45 minutes is compatible with achalasia. The distal oesophageal impedance transit exceeding 5.45 minutes has a positive predictive value of 90.5% for the BS finding achalasia on BS study [5], if the BS test was performed at the time of the follow-up oesophageal physiology study.

The oesophageal transit from z1 to z6 from the first swallow of saline (7.20 seconds), which was actually normal [22,31,34]. This suggests the liquid transit from proximal to distal oesophagus was normal, and the reason for the retention was purely occurring from the resistance at the GOJ (i.e., owing to non-relaxation of LOS and not because of the aperistaltic oesophagus). The subsequent swallowing of saline was filling the oesophagus at the rate of 1.94 cm/seconds (or 116.67 cm/minute) whilst the clearance rate was occurring at 0.69 cm/minute. This led to the oesophageal retention of saline, and the volumetric clearance rate of approximately 4.42 mL passing into the stomach every minute. This may be close to stenosis, which could explain the patient’s dysphagia and weight loss.

## Conclusions

Achalasia is an uncommon disorder, and many physicians may not frequently encounter patients with achalasia to refer them for an oesophageal physiology specialist test. Moreover, the clinical case in this report demonstrated the need for considering oesophageal physiology surveillance in some patients who are suspected of achalasia or low-grade GOJ outflow obstruction (this would be similar to endoscopy surveillance for other GI diseases like Barrett’s oesophagus, recurring GI cancers, and monitoring inflammatory bowel disease).

The features captured on HRM screening for suspecting achalasia were the borderline readings for oesophageal spasmatic activity during standard 5 ml water swallows, intraoesophageal body pressurisation observed during RDC, normal LOS tone, with similar or equal IRP thresholds on standard 5ml water swallows, and significantly reduced reflux events on MII-pH study. Many of the motility features would not be observed on CM to detect the pre-achalasia state. Further research is required to understand the pre-achalasia features on HRM and formulate a pathway to offer patients the oesophageal physiology surveillance. 
